# A Hybrid Model for Safety Pharmacology on an Automated Patch Clamp Platform: Using Dynamic Clamp to Join iPSC-Derived Cardiomyocytes and Simulations of I_k1_ Ion Channels in Real-Time

**DOI:** 10.3389/fphys.2017.01094

**Published:** 2018-01-19

**Authors:** Birgit Goversen, Nadine Becker, Sonja Stoelzle-Feix, Alison Obergrussberger, Marc A. Vos, Toon A. B. van Veen, Niels Fertig, Teun P. de Boer

**Affiliations:** ^1^Division of Heart & Lungs, Department of Medical Physiology, University Medical Center Utrecht, Utrecht, Netherlands; ^2^Nanion Technologies, Munich, Germany

**Keywords:** automated patch clamp electrophysiology, cardiomyocyte, stem cell, dynamic clamp, inward rectifying potassium ion channels, safety pharmacology

## Abstract

An important aspect of the Comprehensive *In Vitro* Proarrhythmia Assay (CiPA) proposal is the use of human stem cell-derived cardiomyocytes and the confirmation of their predictive power in drug safety assays. The benefits of this cell source are clear; drugs can be tested *in vitro* on human cardiomyocytes, with patient-specific genotypes if needed, and differentiation efficiencies are generally excellent, resulting in a virtually limitless supply of cardiomyocytes. There are, however, several challenges that will have to be surmounted before successful establishment of hSC-CMs as an all-round predictive model for drug safety assays. An important factor is the relative electrophysiological immaturity of hSC-CMs, which limits arrhythmic responses to unsafe drugs that are pro-arrhythmic in humans. Potentially, immaturity may be improved functionally by creation of hybrid models, in which the dynamic clamp technique joins simulations of lacking cardiac ion channels (e.g., I_K1_) with hSC-CMs in real-time during patch clamp experiments. This approach has been used successfully in manual patch clamp experiments, but throughput is low. In this study, we combined dynamic clamp with automated patch clamp of iPSC-CMs in current clamp mode, and demonstrate that I_K1_ conductance can be added to iPSC-CMs on an automated patch clamp platform, resulting in an improved electrophysiological maturity.

## Introduction

The Comprehensive *In Vitro* Proarrhythmia Assay (CiPA) initiative aims to find new means of predicting the proarrhythmic risk of newly developed drugs (Gintant et al., 2016), which do not rely exclusively on hERG block, and not on QT prolongation at all. Key aspects are to include results of computer simulations of drug effects on heart rhythm and *in vitro* assays using human stem cell-derived cardiomyocytes (hSC-CMs).

Characterization of the hSC-CM electrophysiological phenotype has so far shown that these CM express most, but not all cardiac ion channels, which has implications for their use in safety pharmacological assays (Jonsson et al., 2012; van den Heuvel et al., 2014). Importantly, the inward rectifying potassium current, I_K1_ (de Boer et al., 2010), that is highly expressed in adult CMs is not, or hardly, expressed by hSC-CMs (Doss et al., 2012; Goversen et al., 2017), while the pacemaker current I_f_ is expressed consistently. As a result, hSC-CMs display a pacemaker-like phenotype, with a depolarized and unstable resting membrane potential, resulting in spontaneous triggering of action potentials. This is an important issue, as the action potential waveform affects the activity and availability of many cardiac ion channels, as these are voltage-sensitive and rely on a negative resting membrane potential between beats to recover from inactivation after an action potential. For example, in hSC-CMs, sodium channel availability during the action potential is minimal, even though the channels are expressed in sufficient levels. As a consequence, we found in a previous study that, except for drugs blocking the hERG channel, none of the tested drugs that are known to be proarrhythmic in adult cardiomyocytes triggered arrhythmias (early-after-depolarizations) in hSC-CMs (Jonsson et al., 2012). More recent studies have found that iPSC-CMs are not sufficiently mature to detect risks associated with inhibition of the late sodium current (Blinova et al., 2017), or peak sodium current (Ando et al., 2017). Interestingly, adenovirus mediated overexpression of I_K1_ channels in iPSC-CM was demonstrated to improve drug responses (Li et al., 2017).

Several approaches are being adopted to improve the electrophysiological phenotype, for instance overexpression of the I_K1_ channel, which has shown promising results (Vaidyanathan et al., 2016). Another, more controllable approach is to use dynamic clamp to add simulated I_K1_ channels to hSC-CMs (Wilders, 2006; Ortega et al., 2017). The essence of dynamic clamp is that a hybrid model is created by connecting a real cell with a computer simulation of (parts of) a cell. For this to work, one needs a computer simulation that is running in real-time—simultaneously with the experiment on the real cell—so there is an instantaneous interaction between the real cell and the simulation. This works well, and has been described by several groups that applied it in manual patch clamp experiments (Bett et al., 2013; Meijer van Putten et al., 2015). Addition of simulated or overexpressed I_K1_ channels results in a stable, more negative resting membrane potential, increased action potential amplitude, and upstroke velocity, thereby bringing the action potential waveform much closer to that of adult human ventricular cardiomyocytes. The expectation is that this approach will result in a more reliable prediction of drug effects, but this hypothesis has yet to be studied systematically (Goversen et al., 2017).

When considering the use of dynamic clamp in safety pharmacology, the low throughput and complex nature of manual patch clamp in combination with dynamic clamp is problematic. Additionally, as noted in the literature (Meijer van Putten et al., 2015), current implementations require the simultaneous use of two computers that both require user interaction during the experiment, which is not very practical. In this study, we have developed a remote-controlled dynamic clamp system with the purpose to couple and integrate it with automated patch clamp devices, in order to increase throughput and develop new predictive assays using hSC-CMs that are in line with the aims of the CiPA initiative. In this study, we demonstrate its application by creating hybrid human cardiomyocyte models by the addition of virtual I_K1_ current to single, suspended human iPSC-CMs, and recording action potentials on an automated patch clamp device.

## Materials and methods

### iPSC-CM culture and dissociation

Differentiated iPSC-derived cardiomyocytes (Cor.4U, kindly provided by Axiogenesis AG, Germany and Cellartis Cardiomyocytes, kindly provided by Takara Bio Europe AB, Sweden) were cultured according to the suppliers' instructions. The cells were dissociated by incubating them for 15–30 min in TrypLE (Gibco) until detached from the surface of the culture flask, and then kept at 4°C for 30 min before pipetting them to individualize the cells.

### Automated patch clamp electrophysiology

Recordings from single iPSC-CMs were done using a Nanion Patchliner automated patch clamp device at 20°C and standard medium resistance NPC-16 chips. After catching an iPSC-CM, obtaining a gigaseal and breaking into whole cell configuration, several experiments were performed.

From a holding potential of −100 mV, current-voltage recordings were made using voltage steps from −80 to 40 mV for 20 ms increasing in 10 mV steps at 2 s intervals (sodium ion currents), and from −40 to 40 mV for 200 ms increasing in 10 mV steps at 5 s intervals (calcium ion currents), with a 100 ms pre-pulse to −40 mV to inactivate sodium ion currents. I_K1_ current were recorded from a holding potential of −40 mV, with voltage steps from −120 to +30 mV for 1,200 ms increasing in 10 mV steps. I_K1_ was blocked by adding 10 μM Ba^2+^, from these recordings we calculated the Ba^2+^-sensitive steady-state current and report these in I-V diagrams.

Next, the recording mode was switched to current clamp and the effect of adding simulated I_K1_ conductance to the iPSC-CM using dynamic clamp was tested. To this end, a current stimulus was optimized for each cell individually to reliably induce action potentials (APs) at a rate of 0.5 Hz. The stimulus was 1 ms long and ranged from 0.6 to 3 nA. The same stimulus was used to trigger APs while exposing the cells to increasing simulated I_K1_ conductance. If the conductance was set too low, no effect on the AP was detectable, if set too high, no AP could be induced. The simulated I_K1_ conductance range varied considerably between individual cells, 200 to 2,000 pS/pF were used across cells.

All experiments were done using extracellular solution containing (in mmol/L) 140 NaCl, 10 HEPES, 5 Glucose, 4 KCl, 2 CaCl_2_, 1 MgCl_2_, pH 7.4 (NaOH), 298 mOsm, and intracellular solution containing 110 KF, 10 KCl, 10 NaCl, 10 HEPES, 10 EGTA, pH7.2 (KOH), 285 mOsm. In some experiments, potassium salts were replaced by cesium salts to expose calcium ion currents otherwise obscured by potassium ion currents. Since potassium currents turned out to be negligible, no difference referring to (real) potassium conductance was observed between those recordings.

### Dynamic clamp system

Experiments were done using a dynamic clamp system developed in house at UMC Utrecht, Utrecht, The Netherlands. The system runs on the Labview RT operating system, and simulates I_K1_ current in response to membrane potential measured from the iPSC-CM. Real-time simulation of the I_K1_ current was done using the model by Ishihara et al. (2009), and simulations were done at 20 kHz, on four independent channels, allowing simultaneous dynamic clamp of four iPSC-CMs. We have included as supplementary information the used I_K1_ model (Supplementary Data Sheet 1), a diagram explaining our dynamic clamp implementation (Supplementary Figure 1) and a flowchart describing the various steps taken during the dynamic clamp experiments (Supplementary Figure 2).

**Figure 1:**
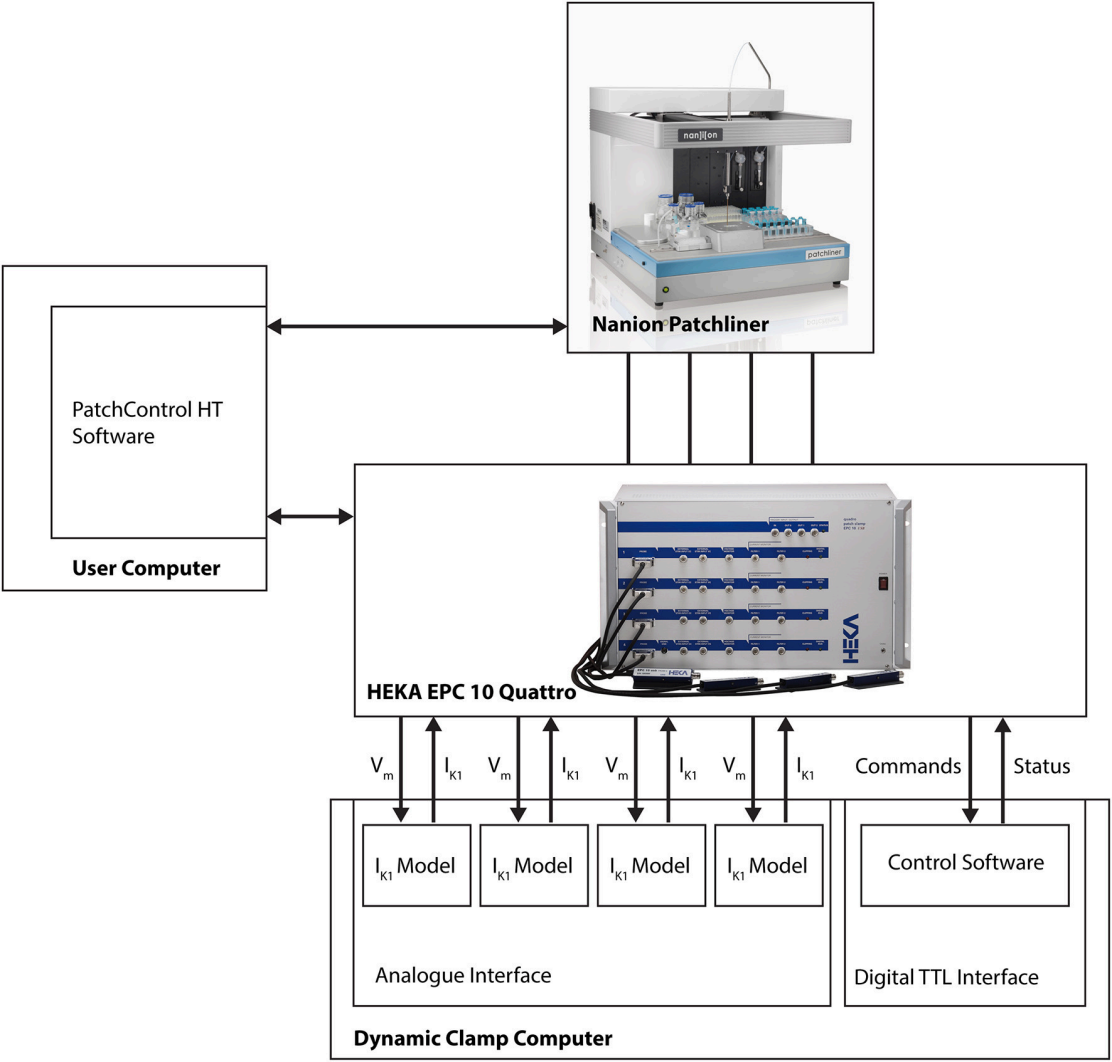
Block diagram showing connections between Patchliner, patch clamp amplifier and dynamic clamp system. For a more detailed depiction of the dynamic clamp system we refer to Supplementary Figure 1.

The dynamic clamp system is coupled to the HEKA EPC-10 Quattro amplifier that is part of the Patchliner setup (see Figure 1). The connections couple the membrane potential of each iPSC-CM with each I_K1_ simulation channel and return the computed I_K1_ current to the iPSC-CM via the external stimulus input of the EPC-10 amplifier. Remote control is achieved via additional couplings that allow bi-directional communication between the dynamic clamp system and the Patchliner setup. For this we use the standard digital input and output channels of the HEKA EPC-10 Quattro amplifier. These are used to continuously read dynamic clamp system status and set model parameters (e.g., I_K1_ conductance, membrane capacitance or external K^+^ ion concentration) when needed. Converting a model parameter to a digital command was done using macros in Patchmaster.

Because of this tight integration with the APC and its software, no direct user interaction with the dynamic clamp setup is necessary. The dynamic clamp system is completely controlled from within the HEKA PatchMaster or the Patchliner PatchControlHT software used to run experiments, and can be set automatically using the programming features in these software programs (i.e., Protocol Editor in PatchMaster or Tree Editor in PatchControlHT).

### Statistics

All results are presented as mean ± standard error of the mean (s.e.m.). Differences in mean outcomes were tested using a One-way ANOVA followed by Tukey's multiple comparisons test, *p*-values smaller than 0.05 were considered significant.

## Results

### Na^+^ and Ca^2+^ currents in iPSC-CMs

Single iPSC-CMs dissociated from iPSC-CM cultures were loaded in the automated patch clamp device and studied in voltage clamp mode to record Na^+^ and Ca^2+^ currents. Capture of the iPSC-CM in the patch clamp chip was efficient, with appropriate seals in 58% of captured cells (see Table 1). After obtaining whole cell configuration, Na^+^ and Ca^2+^ currents could be recorded in ~70% of iPSC-CMs (see Table 2).

**Table 1:** iPSC-CMs can be captured efficiently using automated patch clamp and display Na^+^ and Ca^2+^ currents.

**Capture rate (%)**	**I_Na_ larger than 50 pA (%)**	**I_Ca,L_ larger than 50 pA (%)**
58 (28/48)	71 (20/28)	68 (19/28)

*Six experiments were performed using a total of 3 chips, therefore 48 potential sites on the chip were available and 28 Cellartis iPSC-CMs were captured (with seal resistance >150 MΩ) resulting in a success rate of 58% for capture*.

**Table 2:** Average electrophysiological parameters iPSC-CMs.

**R_Seal_ (MΩ)**	**C_M_ (pF)**	**R_s_ (MΩ)**	**I_Na_ at −30 mV**	**I_Ca,L_ at 10 mV**
			**(nA)**	**(pA)**
976 ± 144 (28)	37 ± 6 (28)	6.0 ± 0.9 (28)	−5.4 ± 1.5 (7)	−157 ± 24 (18)

*Shown are values for seal resistance (R_Seal_), cell capacitance (C_m_) and series resistance (R_s_) for Cellartis Cardiomyocytes captured with seal resistance > 150 MΩ. Na^+^ current at −30 mV and Ca^2+^ current at 10 mV is also shown. Number of cells shown in brackets. Note that the average current is taken from the IV curves and not all cells which had a detectable Na^+^ current were used for the IV analysis*.

**Figure 2:**
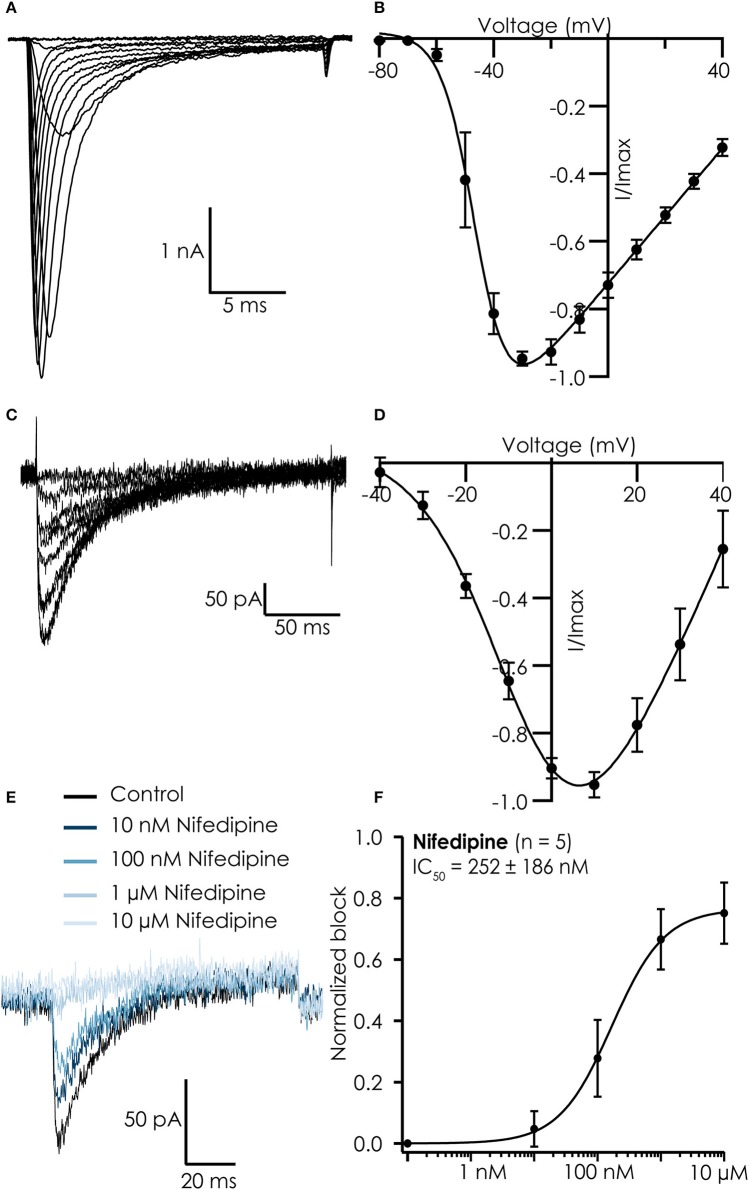
Typical recordings from hiPSC Cellartis Cardiomyocytes recorded on the Patchliner. **(A)** Na^+^ currents in response to increasing voltage steps. **(B)** Corresponding current-voltage plot for an average of 7 cells. A Boltzmann fit revealed a V_half_ of activation of −46 mV. **(C)** Ca^2+^ currents in response to increasing voltage steps. **(D)** Corresponding current-voltage plot for an average of 18 cells. A Boltzmann fit revealed a V_half_ of activation of −5.8 mV. **(E)** Raw traces of Ca^2+^ current in control conditions (black) and after inhibition by increasing concentrations of nifedipine (blue). **(F)** The concentration response curve (normalized to maximum block) for nifedipine for an average of 5 cells. The average concentration response curve was fitted with a standard Hill-equation which revealed an IC_50_ = 252 ± 186 nM (*n* = 5).

Current-voltage relations of both currents were similar to those reported for iPSC-CMs in manual patch clamp experiments (Ma et al., 2011), showing maximal peak current amplitudes at −30 mV for Na^+^ currents (Figures 2A,B) and 10 mV for Ca^2+^ currents (Figures 2C,D). The Ca^2+^ currents could be blocked by nifedipine with an IC_50_ of 252 ± 186 nM, confirming we recorded current conducted by L-type Ca^2+^ channels (Figures 2E,F).

### I_K1_ currents in iPSC-CMs

**Figure 3:**
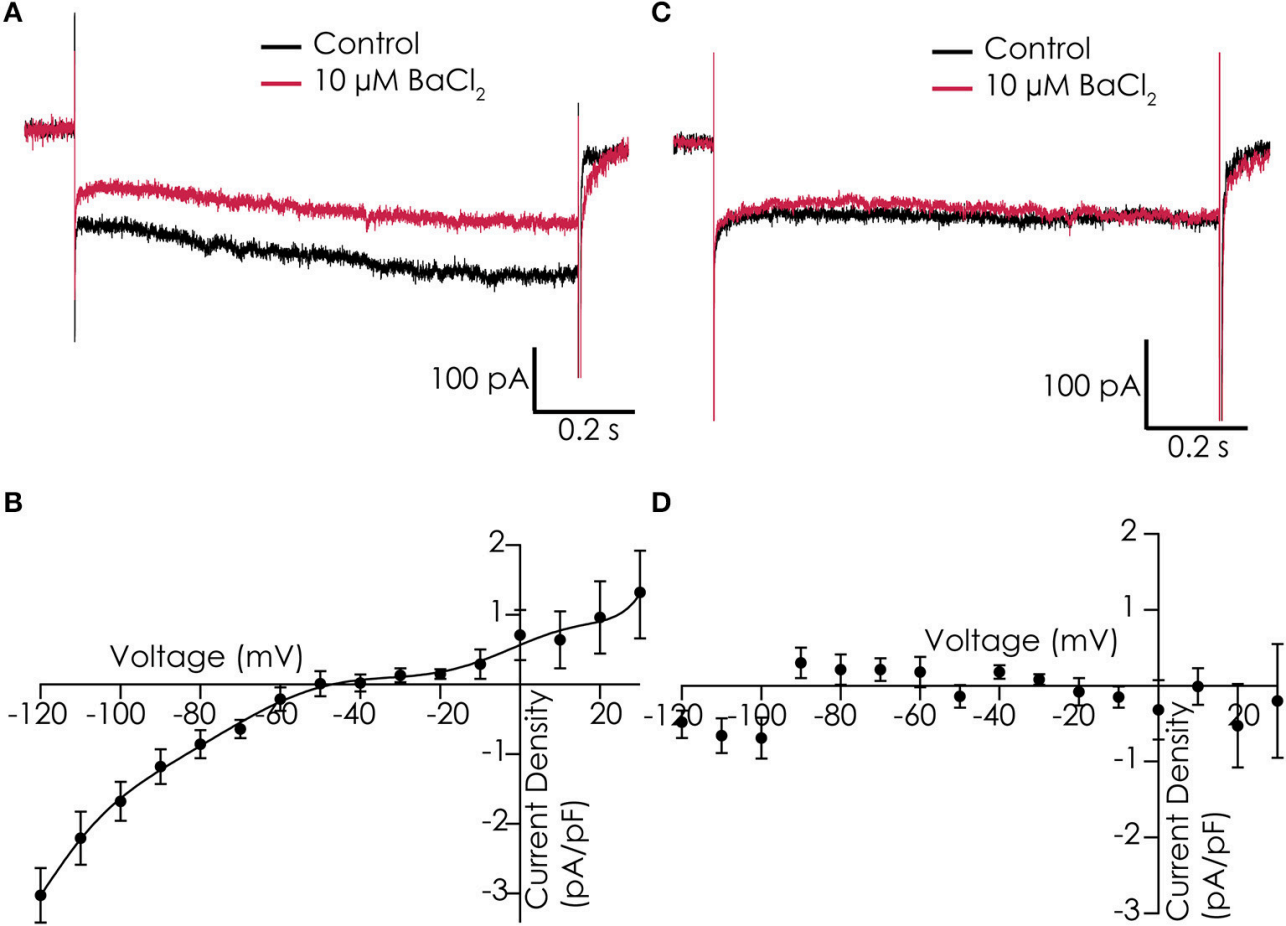
I_K1_ recorded in Cor.4U cells on the Patchliner. **(A)** Example of a cell with I_K1_, shown are responses to a voltage step protocol to −120 mV for 1,200 ms from a holding potential of −40 mV in control conditions (black) and in the presence of 10 μM BaCl_2_ (red). **(B)** Average current-voltage relationship of the Ba^2+^-sensitive current for an average of 7 cells. **(C)** Current traces of an example cell which does not express I_K1_ in control (black) and with 10 μM BaCl_2_ (red). **(D)** Corresponding current-voltage plot of an average of 3 cells showing no Ba^2+^-sensitive current.

Functionality of I_K1_ currents was studied in single dissociated iPSC-CMs that were loaded in the automated patch clamp device. Voltage clamp studies in control solution, and in presence of 10 μM Ba^2+^ to block I_K1_ currents, showed that we could record Ba^2+^-sensitive currents with some resemblance of I_K1_ in 7 out of 12 cells. Examples of cells showing Ba^2+^-sensitive and insensitive currents can be found in Figures 3A,C, respectively. However, current densities were low, and both the reversal potential (which was less negative than expected) and the rectification of the currents were not as observed in adult cardiomyocytes (Figures 3B,D).

### Single suspended iPSC-CMs have depolarized membrane potentials

**Figure 4:**
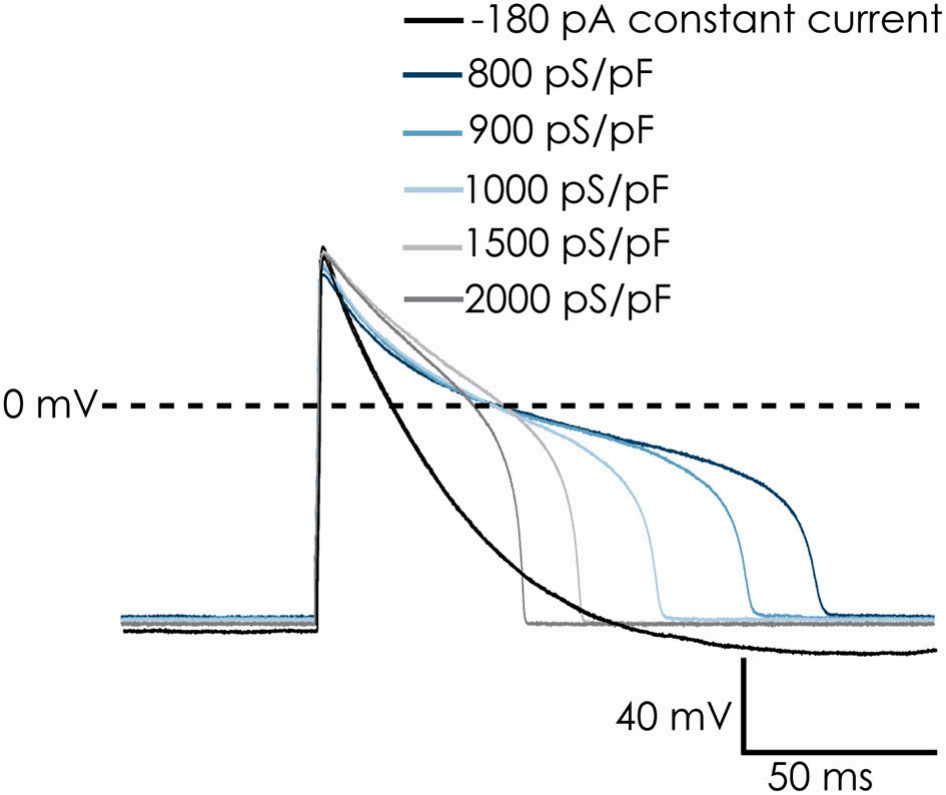
Dynamic clamp used to simulate I_K1_ conductance on action potentials (APs) of Cellartis Cardiomyocytes. The simulated I_K1_ conductance could replace injected current to achieve a native resting membrane potential (RMP) of approximately −94 mV. The cells repolarized faster and the AP duration decreased with increasing I_K1_ conductance. Note: in order to keep a RMP of −94 mV in the absence of simulated I_K1_ conductance, −180 pA of holding current was injected. This was removed upon addition of I_K1_ and RMP remains at −94 mV.

While the voltage clamp experiments demonstrated that the cardiac Na^+^ and Ca^2+^ channels remain functional after dissociation, recording action potentials from iPSC-CMs in suspension was challenging. After switching to current clamp mode, the resting membrane potential of iPSC-CMs was typically between 0 and −15 mV. A negative resting membrane potential close to the reversal potential of K^+^ could be achieved by injecting a small constant, negative holding current. After that, action potentials could be triggered with a brief depolarizing stimulus. However, the action potentials were very short and had a monotonic repolarization without a plateau phase, which does not match well with adult human cardiac action potentials (see Figure 4, black trace labeled “−180 pA”). The I_K1_ channels expressed in fully differentiated human cardiomyocytes hyperpolarize the resting membrane potential of these cells, bringing it close to *E*_*K*_. An important difference with a constant hyperpolarizing current is that I_K1_ channels close upon strong depolarization, allowing development of the plateau phase of the cardiac action potential. In order to improve our methods, and obtain better action potential recordings from suspended single iPSC-CMs, we implemented the dynamic clamp technique on our automated patch clamp device in order to inject simulated I_K1_.

### Hybrid models of suspended iPSC-CMs and simulated I_K1_ channels produce longer action potentials with a plateau phase

Our dynamic clamp implementation is in many ways similar to other published implementations (Bett et al., 2013; Ortega et al., 2014; Meijer van Putten et al., 2015), but differs in two aspects: the functional integration with existing patch clamp control software, and the model used to compute I_K1_. Earlier work has used I_K1_ formulations that model the current as a steady-state current, i.e., without time-dependence. The model by Ishihara et al. used in this study is a more detailed, time-dependent model that includes the rectifying effects of Mg^2+^ and polyamines, as well as two modes of channel closure by spermine (Ishihara et al., 2009). Including this more detailed model is relevant, as time and voltage dependent Mg^2+^ block or unblock can significantly affect action potential duration (Ishihara et al., 2002).

In current clamp experiments with suspended iPSC-CMs, we injected virtual I_K1_ current with varying conductance densities (G_K1_), depending on the specific cell. At depolarized potentials (>−40 mV), the Ishihara model does not generate much outward current, therefore increasing G_K1_ did not immediately induce hyperpolarization. This could be solved by briefly injecting a hyperpolarizing current, bringing the membrane potential to values inducing a sufficiently large outward I_K1_ current. As a result, the membrane potential was maintained at or close to E_K_ (which was −95 mV for the simulated I_K1_ channels) due to the injected virtual I_K1_ current. After this, the constant hyperpolarizing current was switched off, as it was only needed to start the experiment.

**Figure 5:**
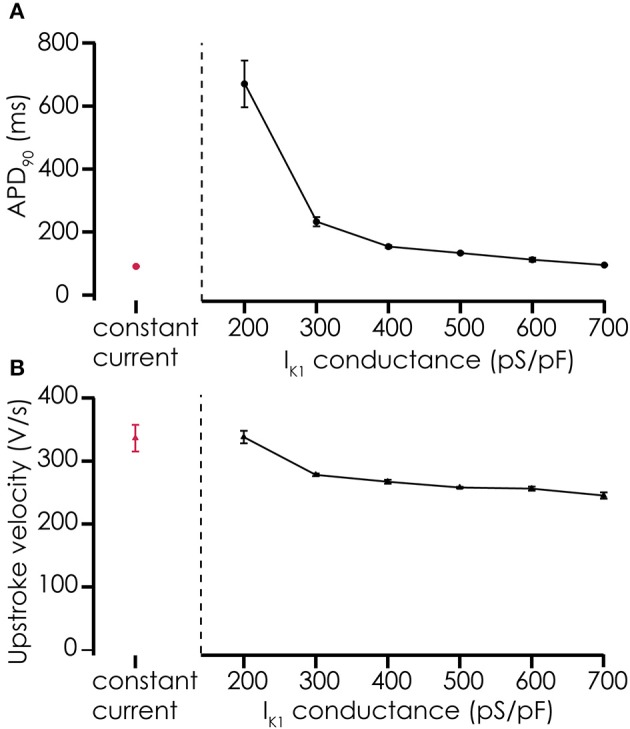
Effects of adding simulated I_K1_ on AP parameters. **(A)** Adding I_K1_ prolongs the APD_90_ of action potentials recorded from Cellartis Cardiomyocytes (*n* = 4) compared to constant current injection. With increasing I_K1_ conductance, the prolongation of the action potential becomes smaller, consistent with the role of I_K1_ in the final repolarization of the cardiac action potential. **(B)** Upstroke velocity of the action potentials after addition of simulated I_K1_ is high, as with constant current injections, and decreases slightly with increasing I_K1_ conductance.

**Figure 6:**
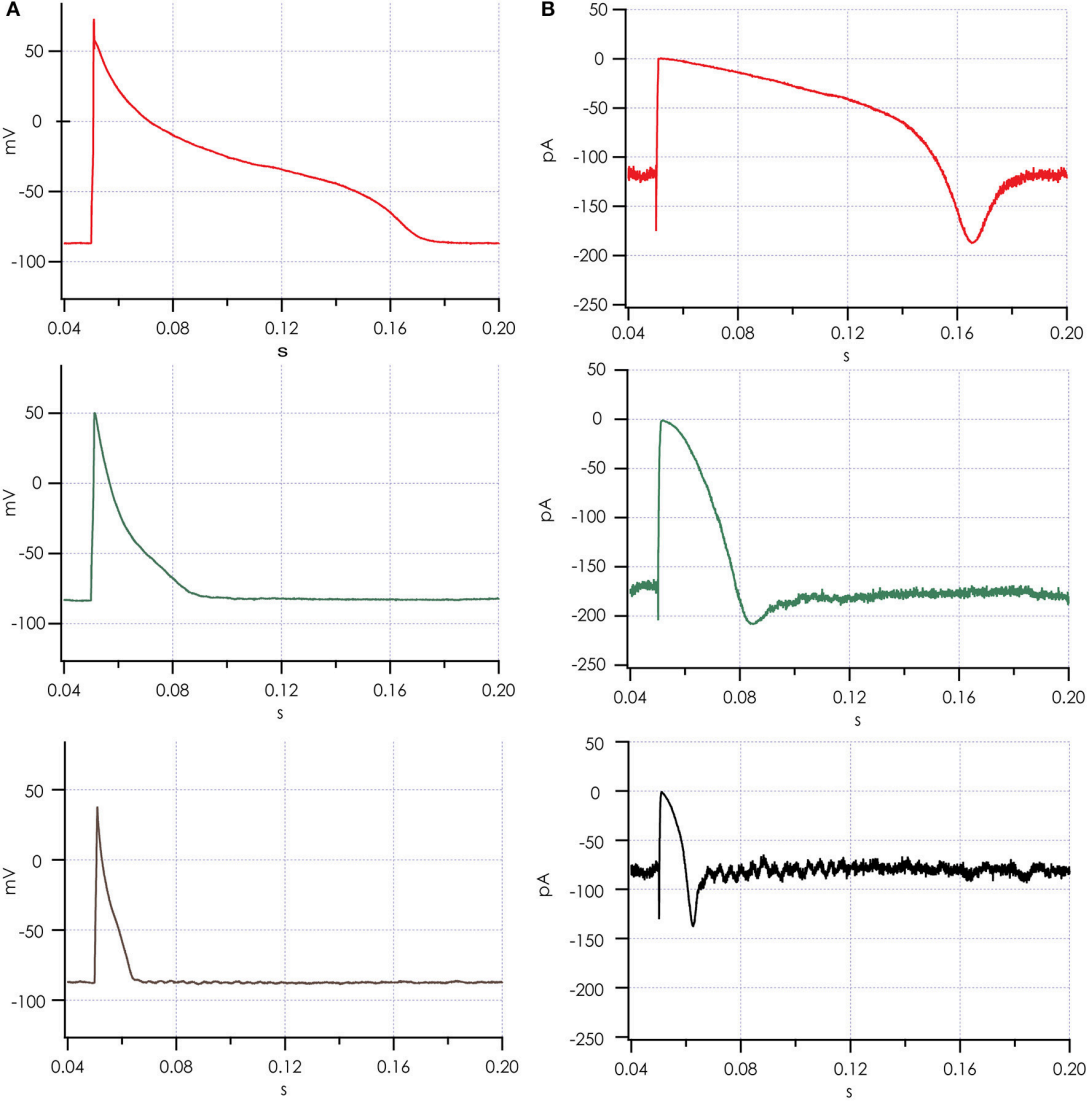
Action potentials recorded simultaneously after adding simulated I_K1_. **(A)** Action potentials from 3 cells recorded in parallel on a Patchliner Quattro. **(B)** Simulated I_K1_ was recorded for each of the 3 cells and is shown. Note the brief increase in I_K1_ during the upstroke of the action potential, where I_K1_ channels respond with increased current to the depolarization induced by the pacing stimulus, which is in line with the slight decrease observed in upstroke velocity with increasing I_K1_ conductance.

Action potentials recorded from iPSC-CMs with addition of virtual I_K1_ differed from the earlier recorded brief and monotonically repolarizing action potentials. Injecting I_K1_ resulted in a stable resting membrane potential, fast upstroke velocities, and prolonged action potential duration with a clear plateau phase (Figure 4). Further increasing G_K1_, resulting in injection of more I_K1_, caused a small additional hyperpolarization, but more significantly, also shortened action potential duration (Figure 5A) and slightly decreased upstroke velocity (Figure 5B). This is consistent with the role of I_K1_ in cardiomyocyte electrophysiology, as it contributes to resting membrane potential stability and the final repolarization phase of the action potential (de Boer et al., 2010). A benefit of our newly developed approach is that experimental throughput can be moderately increased as we can record from, and inject I_K1_ into, 4 cells in parallel. In Figure 6 we show an example of an experiment in which we were able to record APs from 3 iPSC-CM in parallel (Figure 6A) and have also plotted the injected I_K1_ (Figure 6B). The fourth channel was available but for this channel no cell was captured successfully. Additional experiments, in which we studied the effect of G_K1_ on upstroke velocity, showed that we could perform a dynamic clamp experiment in 20 out of 28 wells in 7 experimental runs that used 4 wells per run (71% success rate), see Supplementary Figure 3.

### Addition of virtual I_k1_ channels restores sensitivity of the plateau phase to pharmacological modulation

**Figure 7:**
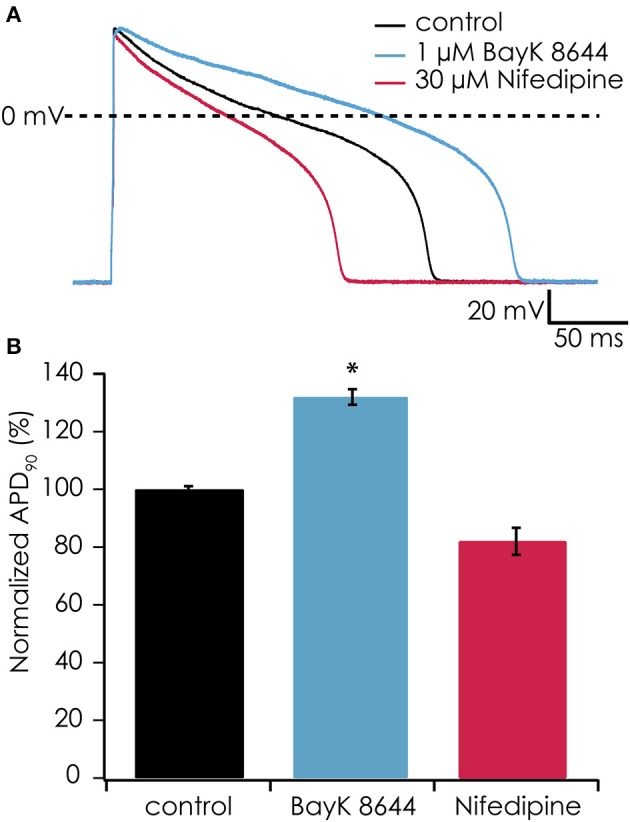
**(A)** The calcium channel agonist BayK-8644 increased AP duration while simulated I_K1_ was added (blue trace). Conversely, the calcium channel antagonist nifedipine decreased AP duration (red trace) as compared with control (black trace). In this experiment, I_K1_ was injected at 400 pS/pF and RMP was −94 mV. **(B)** Average responses from 6 cells showing significantly increased APD_90_ after exposure to 1 μM BayK-8644 (^*^p < 0.05) and decreasing APD_90_ after exposure to 30 μM nifedipine.

In earlier experiments, it proved difficult to observe effects of Ca^2+^ channel antagonists or agonists on the action potential shape of suspended iPSC-CMs when injecting a constant hyperpolarizing current (data not shown), most likely due to suppression of the plateau phase. After observing the restoration of the plateau phase when injecting virtual I_K1_ current, we tested if the action potential became again sensitive to manipulation of the L-type Ca^2+^ current. After injecting I_K1_ (on average G_K1_ was 267 ± 42 pS/pF) we observed an APD_90_ of 118 ± 21 ms (*n* = 6), which significantly prolonged after enhancing the L-type Ca^2+^ channel with 1 μM BayK-8644 to 155 ± 31 ms (*n* = 6, see Figures 7A,B). In contrast, blocking the L-type Ca^2+^ channel with 30 μM nifedipine caused a shortening to 81 ± 14 ms (*n* = 6). These findings demonstrate that enabling the plateau phase of the action potential of suspended iPSC-CMs by addition of virtual I_K1_ channels restores action potential sensitivity to L-type Ca^2+^ channel modulation.

## Discussion

In this study, as a proof of principle, we have demonstrated that the dynamic clamp technique can be used in combination with automated patch clamp devices, thereby creating a higher throughput alternative to manual patch clamp. Using dynamic clamp to add virtual I_K1_ channels to suspended iPSC-CMs allowed us to record action potentials with waveforms that are more representative of the human fully differentiated ventricular cardiomyocyte. This is especially relevant to the CiPA initiative, which aims to use hSC-CMs in drug safety testing.

Achieving higher throughput evaluation of drug effects on action potentials generated by iPSC-CMs will most likely require the use of isolated, suspended cells, as these can be used with automated patch clamp devices. The depolarized resting membrane potential of freshly isolated iPSC-CMs is a challenge that can be overcome, at least to some extent, by using the dynamic clamp technique (this study), but will also require improvement in the dissociation methods used. Dissociation of cardiomyocytes with enzymes disrupting the extracellular matrix, whether native or created in culture, is known to affect the function of I_Kr_, I_Ks_, and I_K1_ channels (Yue et al., 1996; Hoshino et al., 2012). Hoshino et al. demonstrated that the approach used to isolate cardiomyocytes from neonatal mouse hearts has a significant impact on I_K1_ channel function and resting membrane potential. Using enzymatic perfusion of the hearts preserved I_K1_ channels, while the chunk digestion method resulted in four to five times smaller I_K1_ currents and a depolarization of ~20 mV. The approach used in this and other studies to obtain single, suspended iPSC-CMs is very similar to the chunk digestion method, and we have indeed observed only small Ba^2+^-sensitive currents, which may be smaller than those observed in studies using adherent iPSC-CM (Ma et al., 2011; Doss et al., 2012; Nunes et al., 2013). Further improvement of cell dissociation protocols may improve results with iPSC-CMs on automated patch clamp devices. This is supported by recent work by Rajamohan et al. who have used a two-step dissociation protocol and recorded APs from the dissociated iPSC-CMs using both manual and automated patch clamping approaches (same device as used in this study). From the data in this study it appears that the two-step protocol yields cells with a more hyperpolarized membrane potential (Rajamohan et al., 2016).

In the present study, we provide proof-of-principle that automated patch clamp devices and dynamic clamp can be combined successfully. The resulting action potential durations and waveforms are very comparable to those obtained in manual patch clamp experiments in which I_K1_ was added to iPSC-CMs using dynamic clamp (Bett et al., 2013; Meijer van Putten et al., 2015), including the action potential prolongation in response to Bay-K8644. However, more research will be needed to establish the method, and to define its limits and benefits. A better insight into the effects of the specific I_K1_ model that is applied is needed, as well as a well-defined algorithm that allows us to determine which amount of added I_K1_ results in the most predictive results and therefore the best safety pharmacology assay. This should subsequently be demonstrated using the set of drugs defined by CiPA. If these goals can be reached, performing predictive patch clamp experiments with iPSC-CMs with an increased throughput becomes feasible.

## Author contributions

BG: data acquisition, data analysis, data interpretation, revising; NB: data acquisition, data analysis, data interpretation, study design, writing; SS-F, AO: data analysis, data interpretation, revising; TvV, MV, NF: data interpretation, revising; TdB: data acquisition, data analysis, data interpretation, study design, writing, revising. All authors meet the following criteria: (i) Substantial contributions to the conception or design of the work; or the acquisition, analysis, or interpretation of data for the work, (2) Drafting the work or revising it critically for important intellectual content, (3) Final approval of the version to be published, (4) Agreement to be accountable for all aspects of the work in ensuring that questions related to the accuracy or integrity of any part of the work are appropriately investigated and resolved.

### Conflict of interest statement

The authors declare that the research was conducted in the absence of any commercial or financial relationships that could be construed as a potential conflict of interest.

## Supplementary material

Supplementary Figure 1 Diagram describing implementation of dynamic clamping.

Supplementary Figure 2 Flowchart describing steps in dynamic clamp experiment.

Supplementary Figure 3 Influence of I_K1_ conductance on upstroke velocity.

Supplementary Data Sheet 1 CellML implementation of I_K1_ model used in this study.
