# Supplementary figures and images for: A Hybrid Model for Safety Pharmacology on an Automated Patch Clamp Platform: Using Dynamic Clamp to Join iPSC-Derived Cardiomyocytes and Simulations of Ik1 Ion Channels in Real-Time
Source: Front Physiol. 2018 Jan 19;8:1094. doi: 10.3389/fphys.2017.01094 (PMC5782795; doi:10.3389/fphys.2017.01094)

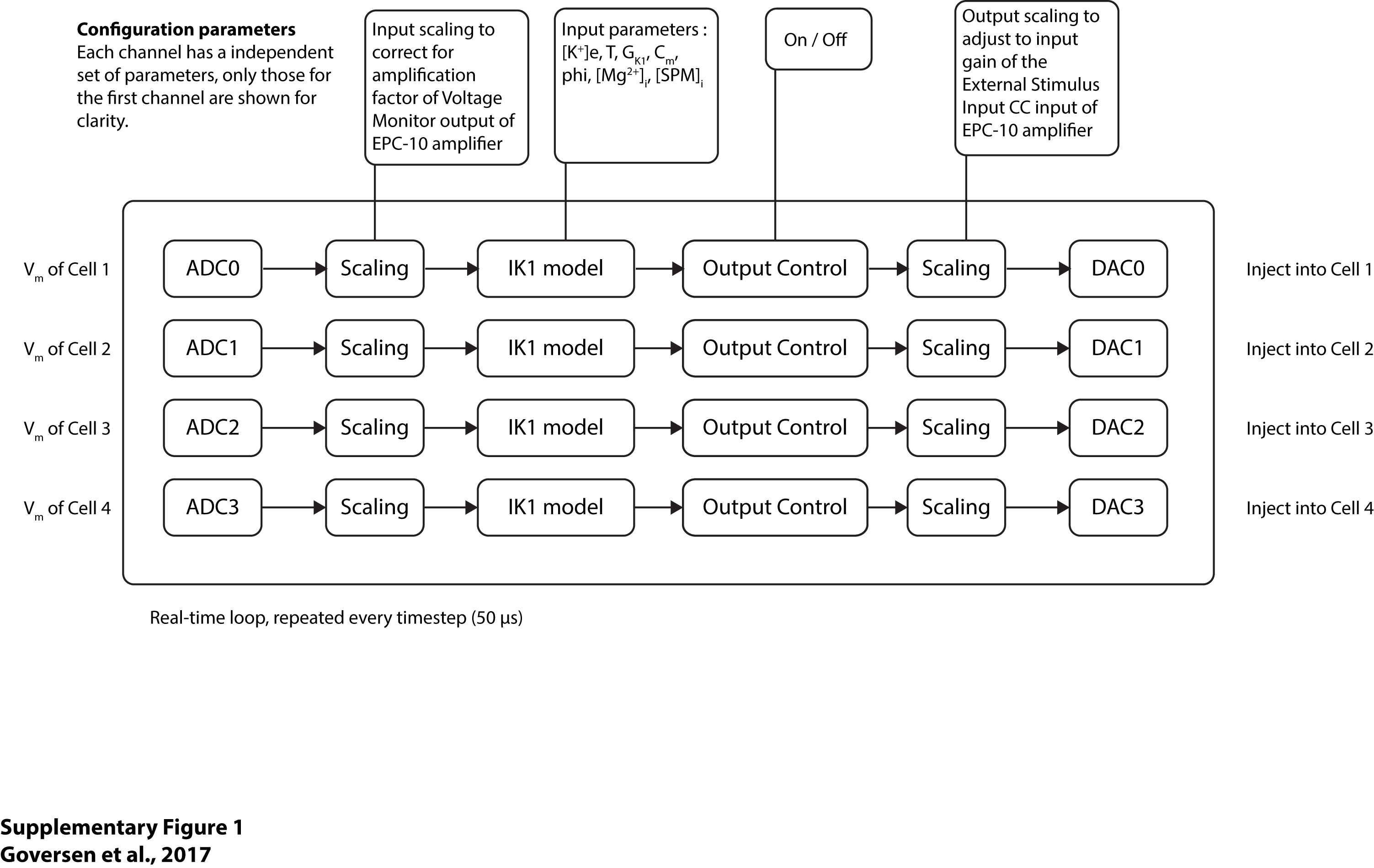

Supplement: Supplementary Figure 1 — Diagram describing implementation of dynamic clamping. [file Image1.TIF]

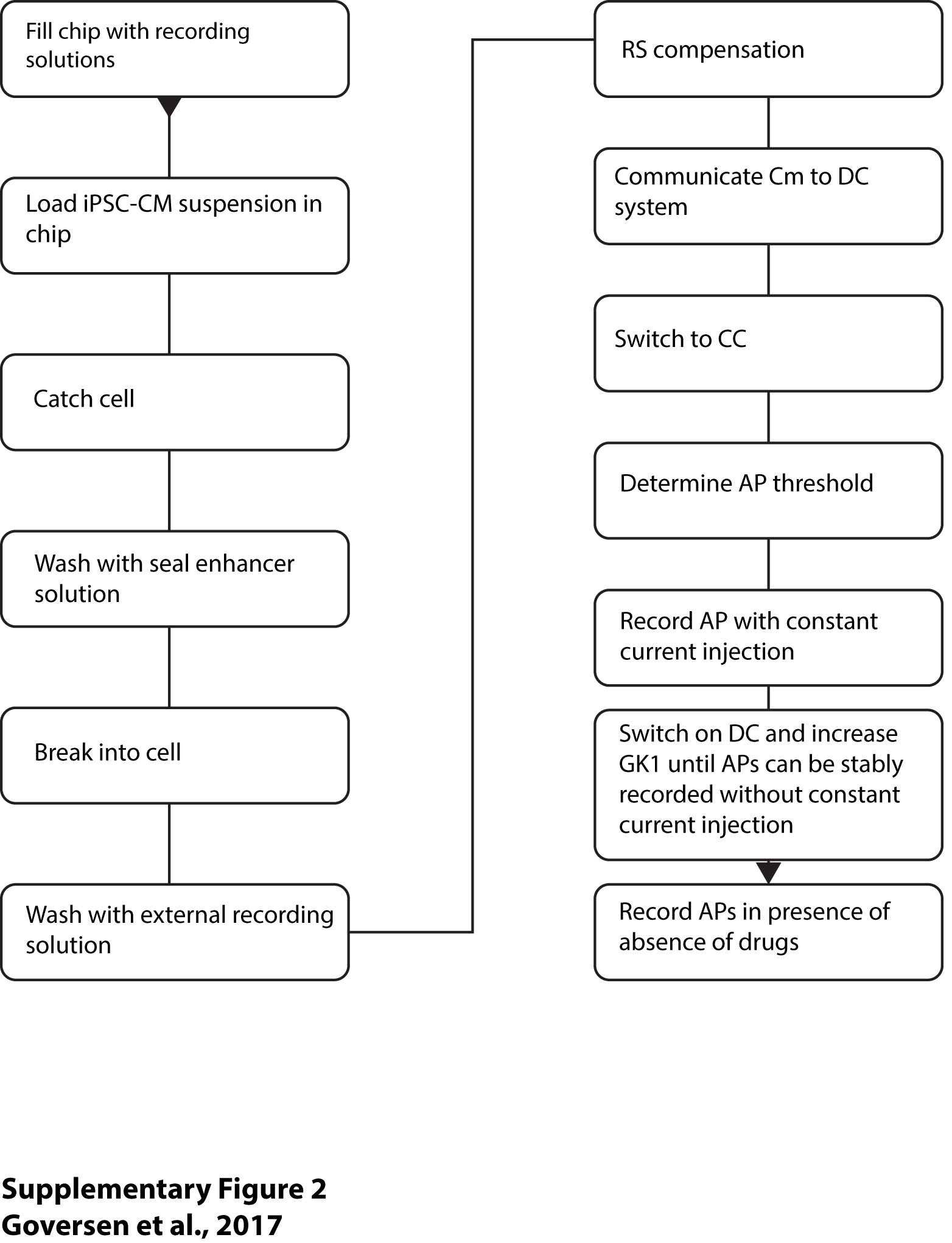

Supplement: Supplementary Figure 2 — Flowchart describing steps in dynamic clamp experiment. [file Image2.TIF]

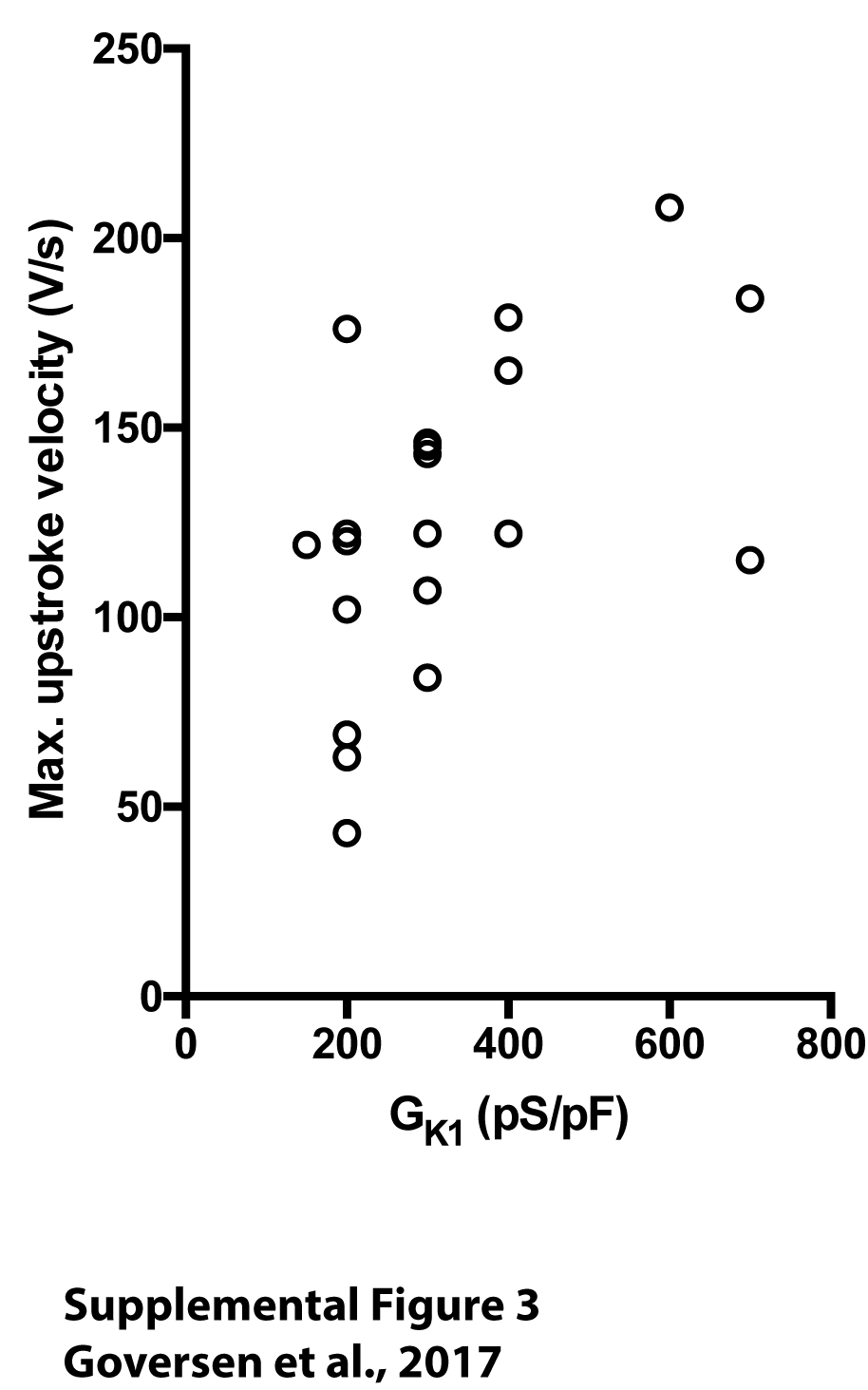

Supplement: Supplementary Figure 3 — Influence of IK1 conductance on upstroke velocity. [file Image3.TIF]
